# Developing and assessing the measurement properties of an instrument to assess the impact of musculoskeletal pain in children aged 9 to 12—the pediatric musculoskeletal pain impact summary score

**DOI:** 10.1016/j.bjpt.2024.101052

**Published:** 2024-03-23

**Authors:** Priscilla Viana da Silva, Steven J. Kamper, Alix Hall, Tie P. Yamato, Lise Hestbaek, Henrik H. Lauridsen, Christopher M. Williams

**Affiliations:** a School of Medicine and Public Health, The University of Newcastle, NSW, Australia; b Hunter New England Population Health, NSW, Australia; c Hunter Medical Research Institute, School of Medicine and Public Health University of Newcastle, NSW, Australia; d Nepean Blue Mountains Local Health District, NSW, Australia; e School of Health Sciences, Faculty of Medicine and Health, The University of Sydney, NSW, Australia; f Priority Research Centre for Health Behaviour, NSW, Australia; g Masters and Doctoral Programs in Physical Therapy, Universidade Cidade de São Paulo, SP, Brazil; h Department of Sports Science and Clinical Biomechanics, Faculty of Health Sciences, University of Southern Denmark, Odense, Denmark; i The Chiropractic Knowledge Hub, Odense, Denmark; j Research and Knowledge Translation Directorate Mid North Coast Local Health District, NSW, Australia

**Keywords:** Children, MSK pain, Pain impact, Patient-reported outcome measures, School-aged

## Abstract

**Background:**

Despite the high prevalence of musculoskeletal (MSK) pain in children, there is a lack of instruments to measure the impact of MSK pain on children's activity and participation.

**Objective:**

To assess the reliability and construct validity of the Pediatric MSK Pain Impact summary score in school children (aged 9 to 12) with MSK pain.

**Methods:**

We used a pragmatic approach in a reflective framework to assess internal consistency, structural validity, convergent validity, and discriminative validity in a sample of 615 children with MSK pain.

**Results:**

The confirmatory factor analysis results indicate that the summary score has limited internal consistency and construct validity. The estimated Cronbach's alpha was 0.63, and most goodness of fit indices met the recommended thresholds (SRMR = 0.030; GFI = 0.993, CFI = 0.955, RMSEA 0.073), although they were close to the lower bounds of the thresholds. The convergent validity showed appropriate correlation of the summary score with quality of life (*r* = -0.33), care-seeking (*r* = 0.45), and medication intake (*r* = 0.37). Discriminative validity showed that the instrument can discriminate between the impact of pain on children with frequent and infrequent (2.93; 95% CI: 2.36 - 3.50) MSK pain.

**Conclusion:**

The Pediatric MSK Pain Impact summary showed limited internal consistency and construct validity; however, it can discriminate between children with frequent and infrequent pain. The results are promising for clinical and research practices as it is a short and convenient tool to be used in school-aged children.

## Highlights

• MSK pain in children and adolescents is highly prevalent, yet instruments are scarce to measure MSK pain outcomes in children.
• The Pediatric MSK Pain Impact summary score is a short and easy instrument to assess MSK pain impact in school-aged children (aged 9 to 12) with MSK pain.
• The result of internal consistency was limited, and the construct validity showed borderline estimates of adequacy. However, the discriminative validity results showed that the instrument differentiates between children with frequent and infrequent MSK pain.

## Introduction

Musculoskeletal (MSK) pain in children is highly prevalent and disabling worldwide.^1^, ^2^, ^3^ Up to 40% of children and adolescents experience persistent MSK pain,^4^ which can substantially impact children's life.^5^ The burden of MSK pain in children results from reduced social interaction with family and friends, increased health care costs, and escalation of anxiety and depression symptoms.^1^^,^^2^ Furthermore, evidence shows that persistent MSK pain in children and adolescents predisposes to MSK pain in adulthood.^3^ While the experience of pain can be broad and cause diverse impacts, children who develop MSK pain often have limited participation in everyday activities.^6^^,^^7^ According to the International Classification of Functioning (ICF), activity and participation are "constituents of health", and encompass individual and societal aspects of functioning.^8^ The impact of pain is defined as the effect of the pain experience on the different aspects of an individual's life and participation.^8^^,^^9^ School and sporting activities are examples of significant components of a child's life that are impacted by the experience of MSK pain.^10^

Despite the potential impact of pain and its high prevalence in children, valid and reliable patient-reported outcome measures (PROMs) that quantify MSK pain impact in children and adolescents are scarce.^11^ Michaleff et al.^11^ compiled a list of the instruments most commonly used to measure pediatric pain. The study found that existing instruments primarily focus on measuring pain intensity, frequency, and location, such as the Faces Pain Scale-Revised (FPS-R) and the Verbal Numerical Rating Scale for pain.^11^ While a few instruments, such as the Young Spine Questionnaire (focused on spinal pain rather than general MSK pain) and The Functional Disability Inventory, measure the impact of pain, they were initially developed and validated for purposes other than MSK pain.^12^^,^^13^

Consequently, instruments validated in adults have been used in children and adolescents to measure MSK pain outcomes, such as the impact of low back pain.^7^^,^^14^ However, using an instrument developed for adults on children without proper validation is not recommended. Children and adults differ significantly in their physical, mental, and social characteristics.^15^ Therefore, it is important to ensure that the instruments used to assess PROMs are valid in this population.^16^ We aimed to develop an instrument using a set of items currently used in research and clinical practice aiming to measure the impact of pain on activity and participation of primary school children (aged 9 to 12) using a reflective framework and assess its reliability (internal consistency) and construct validity.

## Methods

### Design

This study was designed to develop and assess the measurement properties of a tool to measure pain impact in children in grades 4 to 6 (aged 9 to 12) – the Pediatric MSK Pain Impact summary score. The methods of this study were based on the COnsensus-based Standards for the selection of health status Measurement INstruments (COSMIN).^17^ We opted to include the set of items as part of the data collection of an ongoing cluster randomized controlled trial (ACTRN12616001228471), which we considered as a pragmatic approach. The randomized cluster trial was conducted following the Declaration of Helsinki and approved by the Hunter New England Human Research Ethics Committee (Ref. No. 06/07/26/4.04), University of Newcastle (Ref. No. H-2008–0343), and the Maitland-Newcastle Catholic Schools Office. Informed consent was obtained from all parents from all children involved in the study. According to the Committee on Health and Research Ethics, written informed consent has been obtained.

## Development phase

### Definition of the construct

The definition of MSK pain impact for this study was "pain associated with a significant disability"^9^ from the MSK system (including bones, muscles, and joints)^18^ resulting in impairment of the functional status (activity and participation) as per the ICF in primary school-aged children aged 9 to 12.^8^ We proposed a summary score to measure how well the indicators of pain severity; impact on day-to-day activities, on sporting activities, and school absence reflect the construct of MSK pain impact.

### Conceptual framework

We conceptualized the measurement of pain impact in a reflective model as the items (pain severity, activity limitation, limited participation) are common manifestations of pain impact (effect indicator)^11^ and, therefore, are expected to change when the construct (pain impact) changes.^19^^,^^20^

### Item selection process

We collated pre-existing questions typically used to assess the presence of pain, pain severity, and the impact of pain on physical activity, day-to-day activities, and school absence. We included the questions in the baseline survey of a cluster randomized trial that aimed to assess the effectiveness of a school-based physical activity and nutrition intervention (Supplementary material A).^21^^,^^22^ We used the FPS-R to measure pain severity. The FPS-R is a valid instrument to measure pain severity in children aged 5 to 12.^23^, ^24^, ^25^ The FPS-R item responses are presented on a six-point scale ranging from 1 (no pain) to 6 (a lot of pain). We obtained permission from the developers to use the FPS-R as part of this summary score.

To measure activity limitations and participation restrictions following the ICF framework,^8^ we used three pain impact items capturing different aspects of the impact of MSK pain – restrictions in everyday activity, school, and sports. We adapted the wording of items from previous studies on adolescents with LBP to reflect the widespread MSK pain impact on primary school-aged children.^7^ The items asked children to indicate whether their pain caused them to; miss school, stop sports or physical activities, or interfered with everyday activity. Each item was rated on a Likert-type scale with the options; "often", "once in a while", "once or twice", and "never".

### Scoring instructions

The scoring of the Pediatric MSK Pain Impact summary score is a sum of each response option from the four included items: the 6-point Likert pain severity scale (FPS-R) and the three items of pain impact (on 4-point Likert scales). The FPS-R scale score ranges from 1 (no pain) to 6 (a lot of pain). The three pain impact items 4-point Likert scale ranging from 1 (never) to 4 (often). A higher overall Pediatric MSK Pain Score summary score indicates greater functional impact due to MSK pain.

### Validation phase

Classical test theory approach was applied to assess the measurement properties of the Pediatric MSK Pain Impact summary score (Appendix - www.spoergeskemaer.dk/pediatric-msk-impact). We used confirmatory factor analysis (CFA), proposing a one-factor structure, to examine the validity of the four-items-of-pain impact related to functional status.

### Setting

We used baseline data from children enrolled in a cluster randomized trial conducted in 12 Catholic primary (elementary) schools in the Hunter New England Region, New South Wales, Australia. All schools were stratified by size (small, < 300 students; or large, >300 students) and placed in random order. To be eligible, the schools had to have an enrolment of greater than 120 students, be current users of the school mobile communication app (Skoolbag), and not participate in other nutrition or physical activity-based research studies. We aimed to assess the measurement properties of the pain impact questions in children in grades 4 to 6 (aged 9 to 12).

### Participants

All students (aged 9 to 12) attending the 12 schools were invited to participate in the data collection component of the cluster randomized trial via a package sent to their parents, who were asked to provide written consent. Students with parental consent to participate were invited to complete a baseline survey (Supplementary material A). We included students in grades 4 to 6 (aged 9 to 12) who indicated in the baseline survey: 1) the presence of pain or aches in their body, selecting from response options "often," "once in a while," or "once or twice", 2) answered questions relating to the area of their body where they typically experienced pain, and 3) the items about the impact of their pain on their school activities.

### Statistical analysis

a. Item characteristics: We assessed descriptive properties (frequency, means, standard deviations, range, skewness, and kurtosis), the percentage of missing responses for each item, and the use of the distribution of responses for each item. We analyzed each item to assess their feasibility and acceptability to the construct of MSK pain impact. We assessed the correlation between each pair of items to identify possible redundancy. Polychoric correlations were used due to the ordinal nature of the items.^16^^,^^26^ Items with a polychoric correlation > 0.8 were examined for possible exclusion due to high correlation.^27^ Polychoric correlations can be weak (0.2–0.39), moderate (0.4–0.59), strong (0.6–0.79), or very strong (0.8–1).^28^^,^^29^
b. Reliability: We assessed internal consistency as an indicator of the reliability of the questionnaire. Internal consistency is "the degree of the interrelatedness among the items" or the full scale.^16^^,^^20^ We used standardized Cronbach's alpha coefficient (adequate score: ≥ 0.70) and item correlations.^27^^,^^30^ We calculated a standardized Cronbach's alpha due to the differences between the response options for pain severity (six response options) and the impact questions (four response options).
c. Construct validity: We used the following definition: "the degree to which the scores of a measurement instrument are consistent with hypotheses."^16^ We evaluated three indicators of construct validity: structural validity, convergent validity (hypotheses testing), and discriminative validity.
  • Structural validity is the degree to which scores on an instrument adequately reflect the dimensionality of the construct.^16^ A CFA and weighted least-squares in complete cases (students completing the four items) were used to assess the overall adequacy of the model. We estimated the following CFA model fit indices and recommended criteria were estimated: Standardized Root Mean Square Residual (SRMR) < 0.08^31^^,^^32^; Comparative Fit Index (CFI) > 0.95^31^^,^^32^; Root Mean Square Error of Approximation (RMSEA) < 0.06^32^; Bentler-Bonett Normed Fit Index (NFI) > 0.95^31^; Model Chi-squared p-value > 0.05^27^^,^^31^; Goodness of Fit Index (GFI) > 0.90^31^; Adjusted GFI > 0.90^31^; and Parsimonious GFI, for which there is no accepted threshold, but values around 0.5 have been found adequate when other goodness of fit indices are above 0.90.^31^
  • Convergent validity (via hypotheses testing) is the degree to which two measures, that are theoretically related, provide similar results.^33^ We formulated a priori hypotheses about the strength and direction of correlations to examine relationships between the pain impact measurement score and the comparative measures.^16^ Only students who answered all four items were used to calculate the overall score. We used Spearman rank correlation coefficients to assess the relationship between the overall summary score and the comparative measures. The comparative measures, collected at baseline in the trial, were: Pediatric quality of life Inventory (PedsQL) 4.0 total score and physical functioning scale^23^, ^24^, ^25^^,^^34^^,^^35^; care-seeking^7^; medication use^7^; and physical activity and sedentary behavior, both measured by accelerometers.^36^ The description of each comparative measure and each hypothesis is presented in Supplementary material B.
  • Discriminative validity is the degree to which scores on an instrument distinguish differences between known groups.^16^ We evaluated discriminative validity by comparing pain impact scores by groups of students known to differ in frequency of MSK pain episodes. We used mixed linear regression to assess whether students with more frequent episodes of MSK pain scored higher on the Pediatric MSK Pain Impact summary score than those with infrequent pain.^37^ We selected from the included participants the students who experienced pain or aches in their body "often" and reported having pain in the last week (frequent pain). We compared them to those students who responded "once in a while" or "once or twice" and did not report pain in the last week (infrequent pain). The model included a random intercept for schools to account for clustering by school.
  • Sensitivity analyses: We conducted post-hoc sensitivity analyses to compare the primary analysis results for consistency. First, we replicated the primary analysis with only the students who indicated they had pain over one week ("y/n"). We performed a second sensitivity analysis using the same inclusion criteria from the primary analysis but only included students who had experienced pain or aches in their body "often".

## Results

Overall, 815 children aged 9 to 12 from 12 schools returned the baseline survey of the cluster randomized trial. Seven hundred forty-eight children reported whether they had pain or not. Of this, 648 children said they had pain at least "once or twice". Finally, 615 children met our inclusion criteria. A description of the included participants' characteristics is provided in Table 1. Most of the polychoric correlations were weak (< 0.40) for the items "pain intensity", "stayed at home from school", and "stopped doing sports or physical activity" (Table 2). The item "pain interfered with normal activity" had a weak to moderate polychoric correlation. No items illustrated potential redundancy with correlation estimates > 0.80.

**Table 1:** Characteristics of participants *n* = 615.

Characteristics	N (%)
Age (years), mean (SD)	10 (1)
Sex (female), n (%)	327 (53%)
School year, n (%)	
Year 4	223 (36%)
Year 5	211 (34%)
Year 6	181 (29%)
Remoteness classification, n (%)*	
Major city	488 (79%)
Inner/Outer Regional/Remote Australia	127 (21%)
SEIFA disadvantage classification, n (%)	
Most Disadvantaged*	414 (67%)
Least Disadvantaged	201 (33%)
Location of pain, n (%)⊥	
Legs	302 (49%)
Feet	261 (42%)
Head	237 (39%)
Arm	170 (28%)
Spine	128 (21%)
Hips	53 (9%)

⁎ calculated using Social Disadvantage and Accessibility/Remoteness using the Australian Bureau of Statistics 2016 Socio-Economic Index for Australia (SEIFA).

⊥ Proportions may not add up to 100 due to students selecting more than one location.

**Table 2:** Polychoric correlation matrix.

Items	Pain intensity	Stayed at home	Stopped sports/PA	Pain interfered normal activity
Pain intensity	1			
Stayed at home	0.37	1		
Stopped sports/PA	0.25	0.35	1	
Pain interfered normal activity	0.40	0.35	0.44	1

PA, physical activity; Polychoric correlations can be weak (0.2–0.39), moderate (0.4–0.59), strong (0.6–0.79), or very strong (0.8–1).^29^.

**Fig. 1:**
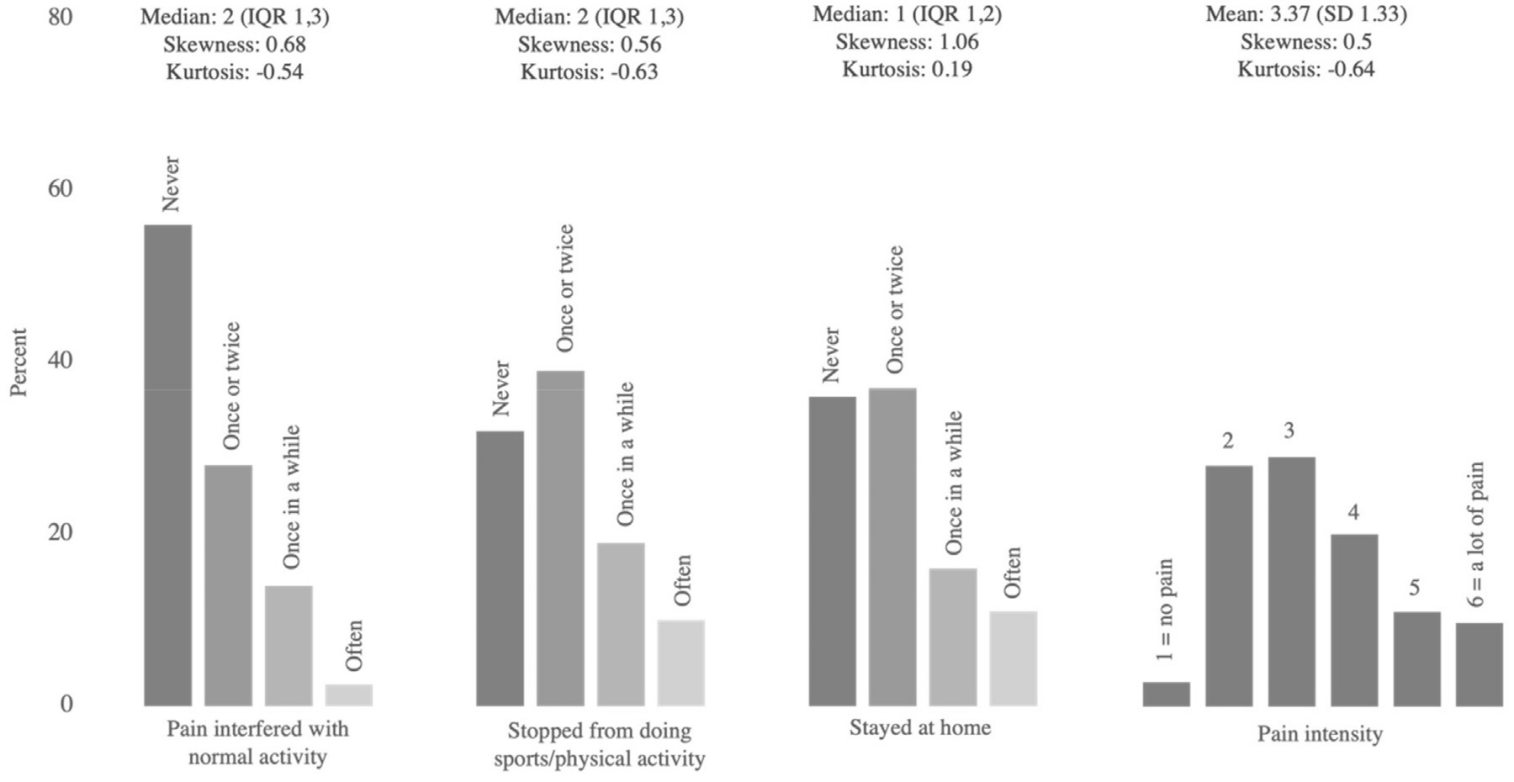
Histogram of each item.

All four items were slightly skewed towards the response item "never" response (positive skewness values, right-hand skew) (Fig. 1). Missing data were minimal (<5%) for all items.

### Reliability

The internal consistency estimate measured by the standardized Cronbach's alpha coefficient was 0.63, below the threshold value of 0.70.^27^^,^^30^

### Construct validity

• *Structural validity:* all but two indices met the recommended criteria for a good fit, with the Chi-squared statistic and Bentler-Bonett NFI below the recommended threshold (Table 3).

**Table 3:** Structural validity indices – confirmatory factor analysis.

Type of Index	Fit index	Fit value	Recommended criteria
Absolute	Pr > Chi-Square†	0.015	> 0.05^31^
	Standardized RMR (SRMR)#	0.030	< 0.08 acceptable^31^^,^^32^
	Goodness of Fit Index (GFI) #	0.993	> 0.90^31^^,^^32^
Incremental	Bentler Comparative Fit Index#	0.955	> 0.95 ideal^31^
	Bentler-Bonett NFI†	0.943	> 0.95^31^
Parsimony	Adjusted GFI (AGFI)#	0.964	> 0.90^31^
	Parsimonious GFI (PGFI)†	0.331	No criteria*^,^^31^
	RMSEA Estimate#	0.073	≤ 0.06^32^

† Indices below the recommended thresholds;.

# Indicative of fair fit;.

⁎ No accepted threshold; however, the penalties applied to this index often result in considerably lower values compared to other goodness of fit indices, PGFI values around 0.5 have been found when other indices are above 0.90^31^; GFI: Goodness of Fit Index; NFI: Normed Fit Index; RMSEA: Root Mean Square Error of Approximation; SRMR: Standardized Root Mean Square Residual.

• *Convergent validity (hypothesis testing):* The total Pediatric MSK Pain Impact summary score was calculated from complete cases (*n* = 599). There were 16 students with at least one missing response on the pain impact items. The correlations with PedsQL 4.0 physical functioning scale (H2), physical activity (H4), and sedentary behavior (H5) did not meet the a priori hypotheses (Table 4). Three out of the six (50%) hypotheses were not confirmed.

**Table 4:** Construct validity hypothesis.

Hypothesis (h)		Correlation		p-value	Confirmed
		Expected	Observed (95% CI)		
H1	PedsQL 4.0 total score	Moderate negative	Moderate negative −0.33 (−0.40, −0.26)	<0.0001	Yes
H2	PedsQL 4.0 Physical Funct	Moderate negative	Weak negative −0.20 (−0.27, −0.12)	<0.0001	No
H3	Care seeking	Moderate positive	Moderate positive 0.45 (0.39, 0.51)	<0.0001	Yes
H4	Medication intake	Moderate positive	Moderate positive 0.37 (0.30, 0.43)	<0.0001	Yes
H5	School Day MVPA	Moderate negative	Weak positive 0.02 (−0.06, 0.10)	0.5410	No
H7	School sedentary behavior	Moderate positive	Weak negative −0.05 (−0.13, 0.03)	0.1139	No

95% CI, 95% confidence interval; H, hypothesis; Physical Funct, physical functioning; r, spearman correlation coefficient. Spearman correlation indices: weak: 〈 0.3, moderate: 0.3 to 0.5, strong: 〉 0.6.

• *Discriminative validity*: 322 students were included in the analysis (122 with frequent pain and 200 with infrequent pain). The discriminative validity showed a mean difference of 2.93 (95% CI: 2.36, 3.50) in the Pediatric MSK Pain Impact summary score between children with frequent and infrequent MSK pain. The percentage of total variance in the Pediatric MSK Pain Impact summary score that could be attributed to the between-school variation was 0.9% (ICC: 0.009 [95% CI: 0.001, 0.127]; *p*<.0001) Students with frequent pain episodes scored 11 (95% CI: 10.5, 11.5), and students with infrequent pain scored 8 (95% CI: 7.7, 8.5) in the Pediatric MSK Pain Impact summary score.

### Sensitivity analysis

The results of both sensitivity analyses (Supplementary material C) did not modify the main findings substantially.

## Discussion

### General findings

We found that the Pediatric MSK Pain Impact summary score had insufficient reliability (internal consistency) and marginal construct validity to assess the impact of MSK pain in children aged 9 to 12. The internal consistency was minimal, with a Cronbach's alpha (0.63) below the recommended criteria (> 0.70).^16^^,^^27^ The polychoric item correlations were adequate. The construct validity may be considered minimal even though estimates met agreed thresholds for most structural validity indices, convergent validity (50% of hypotheses confirmed) and discriminative validity (2.93; 95% CI: 2.36, 3.50). Despite the shortcomings, the instrument showed a reasonable discriminative validity as it could discriminate pain impact levels between children with frequent and infrequent pain.

### Reliability (internal consistency) findings

The Cronbach's alpha (0.63) was below the recommended threshold of 0.70, suggesting limited internal consistency. Therefore, internal consistency for this measure should be interpreted as minimal/emerging.^38^ This is because it is possible that Cronbach's alpha results may be due to the few items of the instrument. Instruments with less than eight items can have lower Cronbach's alpha without necessarily meaning low interrelatedness of the items.^27^^,^^30^ Also, greater Cronbach's alpha estimates are expected in larger samples, meaning that small changes are less likely to affect the overall score, and because our sample is large (*n* = 815), our confidence in the internal consistency result remains fairly strong.^39^ Nevertheless, future investigation into the internal consistency of the measure should be considered.

### Construct validity findings

The analysis of structural validity suggests that the summary score reflects the unidimensionality of the construct of pain impact in school-aged children (aged 9 to 12). It is considered best practice to report at least three goodness-of-fit indices, such as SRMR, RMSEA, and CFI.^16^^,^^31^^,^^32^ In our study, the goodness-of-fit indices met the recommended criteria. However, we acknowledge that most indices were very close to the recommended threshold, which may still be insufficient compared to other measures.^27^

The Pediatric MSK Pain Impact summary score showed moderate convergent validity but poor divergence with other constructs. Two of the three expected positive correlations (care seeking and medication intake) met our pre-specified correlation threshold. Whereas one of three expected negative correlations (quality of life) met the pre-specified threshold. However, physical function showed only a weak negative correlation. The remaining two hypotheses were rejected due to a weak unexpected positive correlation (physical activity) and an unexpected negative correlation (sedentary behavior). While this unexpected cross-over (or lack of) with other constructs may undermine the Pediatric MSK Pain Impact summary independence as a construct, we found the score to have good discrimination of "known group" who would be expected to have a higher burden of pain.^40^

### Limitations

We acknowledge the limitations of our study. First, we opted for a pragmatic approach to develop and assess the measurement properties of the instrument. Following the recommended development steps^19^ could have provided valuable insights for item reformulation or additions to the instrument items. We selected and adapted pre-existing items used in low back pain studies^7^ and embedded the items into an existing trial without conducting pilot and field-testing steps. Additionally, the skewness observed in the response items may suggest the presence of a potential floor effect at the item level, which possibly reflects limited content validity and is likely to limit the potential responsiveness of the measure. Due to feasibility and capacity constraints, we were unable to evaluate additional measurement properties such as Standard Error of Measurement (SEM), Minimal Detectable Change (MDC), responsiveness, and Minimal Clinically Important Difference (MCID). Future research should consider these aspects to provide a more comprehensive evaluation of the instrument's performance.

### Future perspectives for research

Further research should be conducted to expand the applicability of the instrument. First, future studies could improve the content validity of the instrument by asking the perspectives of children, parents, and teachers about the relevance, comprehensiveness, and comprehensibility of the items, response options, and instructions. Furthermore, future research should investigate how emotional, cognitive, and social aspects of pain can be integrated into this short instrument, as these aspects can potentially affect children's activity and participation.^6^ However, it is important to emphasize that while considering adding new items to the instrument in the future, maintaining its brevity is important for reducing respondent burden while ensuring reliable and valid estimates. Lengthy outcome measures can be particularly hard for children to complete.^11^ Finally, it is recommended to assess the measurement properties of this instrument in other age ranges. As children's cognitive development evolves with age, their perceptions of subjective pain constructs may differ. Exploring this across various age groups will yield valuable insights into the measure's validity and applicability across the developmental spectrum.^41^^,^^42^

## Conclusions

The Pediatric MSK Pain Impact summary score is a short instrument designed to assess the impact of MSK pain on the physical functioning of school-aged children aged 9 to 12. The instrument could distinguish between children with infrequent and frequent pain who are likely to be impacted by MSK pain. However, the results showed limited internal consistency and moderate convergent validity, as only three hypotheses were met. It is possible that the short length of the instrument might have negatively impacted the measurement properties. Further research should investigate additional measurement properties and whether adding extra items or reformulating current ones improves the instrument's performance. We also note that the measure focuses on the physical impact domain and does not address other domains, such as psychological well-being and social participation.

## Data Availability Statement

The data supporting this study's findings are not publicly available and will be available upon reasonable request and with the permission of all involved authors.

## Author contributions

Conceptualization, P.V.S., C.M.W., S.J.K., L.H., H.H.L., and A.H.; Methodology, P.V.S., S.J.K., C.M.W., L.H., H.H.L., and A.H.; Software, A.H.; Validation, A.H., L.H. and H.H.L.; Formal analysis, A.H., (C.L.); Investigation, N.N., and N.M,.; Resources, N.N., N.M., S.J.K., and C.M.W; Data curation, A.H., N.M., and N.N.; Writing—original draft preparation, P.V.S., T.P.Y, S.J.K., and C.M.W.; writing—review and editing, P.V.S., S.J.K., L.H., A.H., H.H.L, T.P.Y., and C.M.W.; Visualization, P.V.S., and C.M.W.; Supervision, S.J.K., T.P.Y., C.M.W., L.H., and H.H.L.; Project administration, N.N, N.M., C.M.W.; Funding acquisition, N.N., L.W. All authors have read and agreed to the published version of the manuscript.

## Funding

This research did not receive any specific grant from the public, commercial, or not-for-profit funding agencies. Hunter New England Health, Population Health supported the infrastructure of this trial.

## Declaration of competing interest

The authors declare no conflict of interest.

## Appendix Supplementary materials

Image, application 1
