# Supplementary material for: Developing and assessing the measurement properties of an instrument to assess the impact of musculoskeletal pain in children aged 9 to 12—the pediatric musculoskeletal pain impact summary score
Source: Braz J Phys Ther. 2024 Mar 23;28(2):101052. doi: 10.1016/j.bjpt.2024.101052 (PMC11039311; doi:10.1016/j.bjpt.2024.101052)
Supplement: Supplementary file 1 [file mmc1.pdf]

Supplementary material A – Main trial Baseline Survey

GOOD FOR KIDS, GOOD FOR LIFE

School Student Questionnaire
Version 2 dated 20/01/2017

Thank you for helping us today.
Many students throughout the Hunter New England area are helping us by completing this questionnaire.
There are no right or wrong answers. Please answer all the questions the best that you can in the space provided.
This questionnaire will help us understand more about the health of young people.
The answers are confidential and will be looked at by the survey team and no-one else.
No-one at your school will see your answers.
Thank you again for being part of this important survey!

Section 1: DEMOGRAPHICS

We will first ask a few questions about you…

**Q1**	What is your first name?	________________________________
**Q2**	What is your last name?	________________________________
**Q3**	What year are you in? *(circle ONE number only)*	1. Year 4<br>2. Year 5<br>3. Year 6
**Q4**	What class are you in?	____________
**Q5**	What school do you go to?	________________________
**Q6**	What is your date of birth?	______ /______/__________ (Day/Month/Year)
**Q7**	Are you a boy or a girl? *(circle ONE number only)*	1. Boy<br>2. Girl
**Q8**	What suburb do you usually live in?	________________________
**Q9**	What is the postcode where you usually live?	__ __ __ __

Section 2: THE FOODS YOU EAT

The next set of questions is about the foods that you eat

		Per week	Per day

Please select how often you usually eat the following foods.<br>*(circle ONE number only for each question)*		Never or Rarely	1-2 times per week	3-4 times per week	5-6 times per week	1 time per day	2 or more times per day
**Q10**	Potato crisps or pretzels?	0	1	2	3	4	5
**Q11**	Chocolate?	0	1	2	3	4	5
**Q12**	Lollies?	0	1	2	3	4	5
**Q13**	Muesli bars or fruit bars?	0	1	2	3	4	5
**Q14**	Savoury biscuits, such as shapes, jatz or saos?	0	1	2	3	4	5
**Q15**	Sweet biscuits, such as tiny teddies or timtams?	0	1	2	3	4	5
**Q16**	Ice cream or ice blocks?	0	1	2	3	4	5
**Q17**	Hot chips?	0	1	2	3	4	5
**Q18**	Pies/sausage rolls/other savoury pastries?	0	1	2	3	4	5
**Q19**	Hot dogs?	0	1	2	3	4	5
**Q20**	Pizza?	0	1	2	3	4	5
**Q21**	Cakes, donuts, iced buns and muffins?	0	1	2	3	4	5
**Q22**	Noodle snacks?	0	1	2	3	4	5
**Q23**	Cheese and/or bacon rolls or vegemite scrolls?	0	1	2	3	4	5

Please select how many cups of the following drinks you usually consume.<br>*(circle ONE number for each question)*		Per week			Per day	
		1 cup or less per week	2-4 cups per week	5-6 cups per week	1 cup per day	2 or more cups per day
**Q24**	**Fruit Juice** (1 cup = 250ml, a household tea cup or 1 large popper)	1	2	3	4	5
**Q25**	**Water (tap or bottled)** (1 cup= 250ml, a household tea cup; 1 average bottle of water = 2 ½ cups)	1	2	3	4	5
**Q26**	**Soft drink, cordials or sports drink, such as lemonade or Gatorade** (1 cup = 250ml; 1 can of soft drink = 1 ½ cups)	1	2	3	4	5
**Q27**	**'Diet' soft drink or diet cordial, such as Diet Coke, Diet Sprite or Coke Zero** (1 cup = 250ml; 1 can of soft drink = 1 ½ cups)	1	2	3	4	5
**Q28**	**MILK** (1 cup = 250ml, a household tea cup) **Include all types of milk including flavoured milk and milk on cereal**	1	2	3	4	5

Section 3: GENERAL HEALTH AND WELLBEING

The next section will ask about a list of things that might be a problem for you. Please tell us how much of a problem each one has been for you during the past ONE month by circling:

0 if it is never a problem
1 if it is almost never a problem
2 if it is sometimes a problem
3 if it is often a problem
4 if it is almost always a problem

There are no right or wrong answers.

**In the past ONE month, how much of a problem has this been for you…** *(circle ONE number only for each question)* **About my health and activities** (problems with…)		Never	Almost Never	Sometimes	Often	Almost Always
**Q29**	It is hard for me to walk more than one block	0	1	2	3	4
**Q30**	It is hard for me to run	0	1	2	3	4
**Q31**	It is hard for me to do sports activity or exercise	0	1	2	3	4
**Q32**	It is hard for me to lift something heavy	0	1	2	3	4
**Q33**	It is hard for me to take a bath or shower by myself	0	1	2	3	4
**Q34**	It is hard for me to do chores around the house	0	1	2	3	4
**Q35**	I hurt or ache	0	1	2	3	4
**Q36**	I have low energy	0	1	2	3	4
**In the past ONE month, how much of a problem has this been for you…** *(circle ONE number only for each question)* **About my feelings** (problems with…)		Never	Almost Never	Sometimes	Often	Almost Always
**Q37**	I feel afraid or scared	0	1	2	3	4
**Q38**	I feel sad or blue	0	1	2	3	4
**Q39**	I feel angry	0	1	2	3	4
**Q40**	I have trouble sleeping	0	1	2	3	4
**Q41**	I worry about what will happen to me	0	1	2	3	4

**In the past ONE month, how much of a problem has this been for you…** *(circle ONE number only for each question)*<br><br>**How I get along with others** (problems with…)		Never	Almost Never	Sometimes	Often	Almost Always
**Q42**	I have trouble getting along with other kids	0	1	2	3	4
**Q43**	Other kids do not want to be my friend	0	1	2	3	4
**Q44**	Other kids tease me	0	1	2	3	4
**Q45**	I cannot do things that other kids my age can do	0	1	2	3	4
**Q46**	It is hard to keep up when I play with other kids	0	1	2	3	4

**In the past ONE month, how much of a problem has this been for you…** *(circle ONE number only for each question)*<br><br>**About school** (problems with…)		Never	Almost Never	Sometimes	Often	Almost Always
**Q47**	It is hard to pay attention in class	0	1	2	3	4
**Q48**	I forget things	0	1	2	3	4
**Q49**	I have trouble keeping up with my schoolwork	0	1	2	3	4
**Q50**	I miss school because of not feeling well	0	1	2	3	4
**Q51**	I miss school to go to the doctor or hospital	0	1	2	3	4

Section 4: ACHES AND PAINS
The last section of the questionnaire asks about aches and pains in your body

**Q52**	Have you had pains or aches in your body? *(circle ONE number only)*	1 Often<br>2 Once in a while<br>3 Once or twice<br>4 Never – *If you answered never, you do not need to answer any more questions. Thank you for your time!*
**Q53**	Please mark on this picture with an 'X' where you typically feel these pains and aches:	

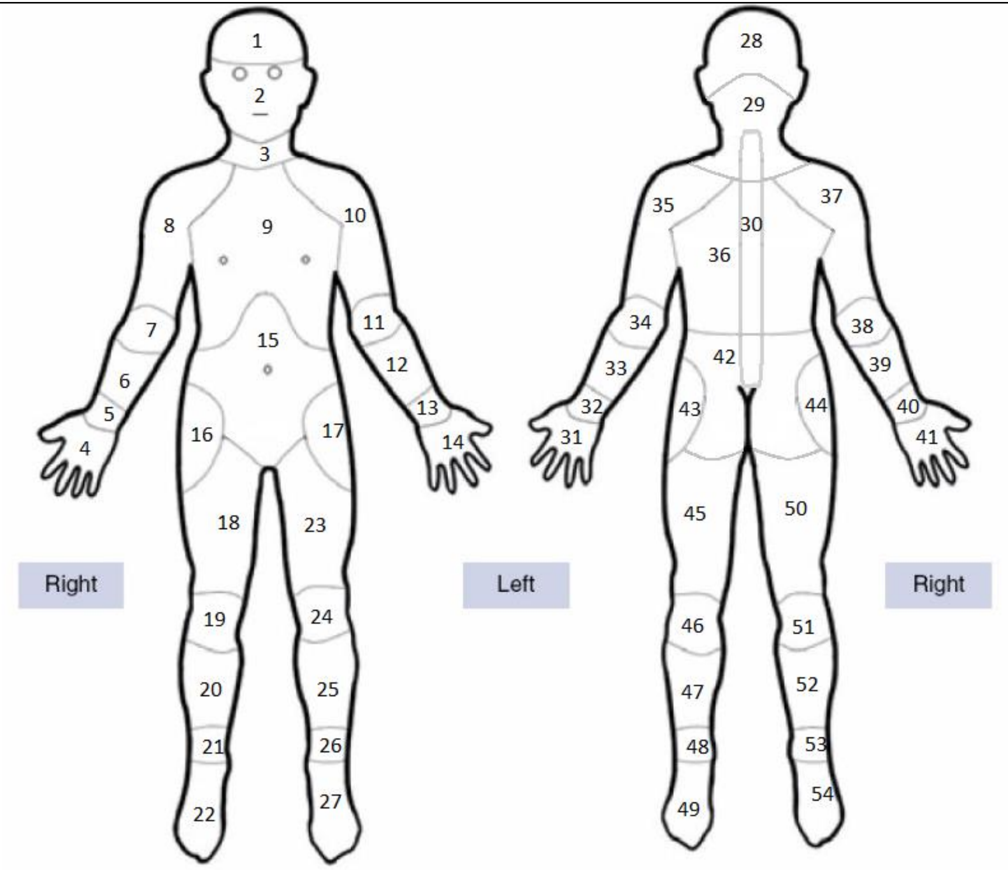

**Q54**	The faces below show how much something can hurt. The pain ranges from "No pain" to "A lot of pain" (cross _ONE_ answer only)<br>Put a cross (X) on the face which shows how much pain you have had when it was worst

No pain					A lot of pain
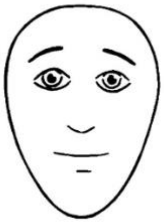	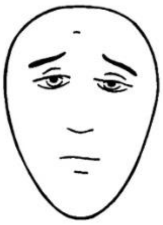	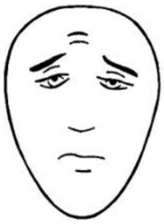	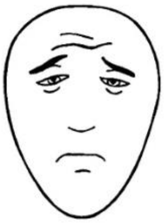	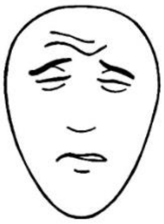	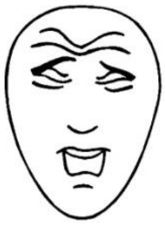
1	2	3	4	5	6

**Q55**	How did the pain start?<br>*(circle _ONE_ circle only)*	1 Accident or injury<br>2 Gradual onset with no injury<br>3 Don't know/unable to remember
**Q56**	Have you had pains or aches in your body **in the last week?**<br>*(circle _ONE_ circle only)*	1 Yes<br>2 No
**Q57**	Have you had pains or aches in your body **today?**<br>*(circle _ONE_ circle only)*	1 Yes<br>2 No

**Q58**	Have you stayed home from school because of the pain?<br>*(circle ONE circle only)*	1 Often<br>2 Once in a while<br>3 Once or twice<br>4 Never
**Q59**	Has your pain sometimes stopped you from doing sports or physical activities (e.g. running, cycling)?<br>*(circle ONE circle only)*	1 Often<br>2 Once in a while<br>3 Once or twice<br>4 Never
**Q60**	Have you been to a doctor, chiropractor or physiotherapist because of the pain?<br>*(circle ONE circle only)*	1 Often<br>2 Once in a while<br>3 Once or twice<br>4 Never
**Q61**	Have you taken any medicine to relieve the pain?<br>*(circle ONE circle only)*	1 Often<br>2 Once in a while<br>3 Once or twice<br>4 Never
**Q62**	Has your pain interfered with your normal activity?<br>*(circle ONE circle only)*	1 Often<br>2 Once in a while<br>3 Once or twice<br>4 Never

This is the end of the questionnaire. Thank you for completing this survey!

This information will allow us to make programs to help students stay active and health.

Supplementary material B – Comparative measures

Comparative Measures	Comparative measure description	Hypothesis	
**PedsQL 4.0** [1,6,12,13]	The PedsQL 4.0 is a valid and reliable 23-item instrument to measure health-related quality of life in paediatric populations. We used child self-report for ages 8 to 12 years. This scale comprises physical, emotional, social, and school functioning subscales, measured over the past month, each item is scored on a 5-point scale.	H1 – The PedsQL 4.0 total score measures children' s physical, emotional, and social wellbeing. Higher scores indicate higher quality of life among healthy children compared to children with a chronic health condition.[1] We expected a negative and moderate (0.3 to 0.6) correlation between pain impact and the PedsQL 4.0 total score.[1,6]	H2 – The PedsQL 4.0 physical functioning scale reflects the physical function consequences of chronic diseases in children.[1-3] Pain can considerably decrease children' s physical function restricting them in their activities (i.e., school and sports).[3,4] We expected a negative and moderate (0.3 to 0.6) correlation between the pain impact score and the PedsQL physical functioning scale.
**Care seeking and Medication intake** [8]	We measured care seeking via students' answers to the following question:<br>' *Have you been to a doctor, chiropractor, or physiotherapist because of your pain?* ' .<br>We measured care seeking via students' answers to the following question:<br>' *Have you taken any medicine to relieve the pain?* ' .<br>For each question students had four options: ' *often* ' , ' *once in a while* ' ; ' *once or twice in total* ' ; or ' *never* ' .	H3 – Wilson and Cleary conceptual model defines symptom as ' a patient' s perception of an abnormal physical, emotional, or cognitive state' that drives patients to seek medical attention in order to understand the origin of the symptom.[7] We expected a positive and moderate (0.3 to 0.6) correlation between pain impact and care seeking.[8]	H4 – Pain in children aged 9 to 12 is more likely to be managed with medication than in younger children (< 9 years).[2,3] We expected a positive and moderate (0.3 to 0.6) correlation between pain impact and whether students have taken medication to relieve their pain.
**Physical activity and sedentary behaviour**	The students' physical activity* was measured using accelerometer for 5 days during school hours (9am to 3pm), distributed daily by the teachers to children as they entered the class for the day and removed at the end of the day. Only students that wore accelerometers ≥80% of the school day, for a minimum of 3 school days were included in the analyses. Accelerometer data were classified as sedentary, light-intensity physical activity, and moderate to vigorous physical activity (MVPA) using the cut-points developed by Chandler et al. [9]	H5 – Guidelines recommend that children and adolescents (aged 6 to 18) perform at least 60 minutes of MVPA each day.[9-11] However, pain can decrease children participation in PA, decreasing the minutes of MVPA considerably. [10] We expected to find a negative and moderate (0.3 to 0.6) correlation between pain impact and the mean daily minutes of MVPA performed during the school day.	H6 – Children in pain are less likely to engage in physical activity during school time.[5] One possible explanation is that children are told to not participate in physical activities in school (i.e., avoid sports) to not aggravate their pain symptoms, reinforcing sedentary behaviour during school-day. We expected to find a moderate positive correlation ($0.30 \leq r < 0.60$) between the questionnaire score of those children in pain and their levels of school-day sedentary behaviour.

*Minutes per day spent in moderate to vigorous physical activity; # ActiGraph GT3X+ (ActiGraph Corporation, Pensacola, FL),
Two research assistants visited each class and demonstrated how to fit the accelerometer to the wrist of their non-dominant hand and answered any questions.

REFERENCES

1. Varni JW. Scaling and scoring of The PedsQL. Accessed 18 Aug 2022. https://www.pedsql.org/PedsQL-Scoring.pdf.
2. Henschke N, Harrison C, McKay D, et al. Musculoskeletal conditions in children and adolescents managed in Australian primary care. *BMC Musculoskelet Disord.* 2014;15:164. 10.1186/1471-2474-15-164.
3. Kamper SJ, Williams CM. Musculoskeletal Pain in Children and Adolescents: A Way Forward. *J Orthop Sports Phys Ther.* 2017;47(10):702-704. https://doi.org/10.2519/jospt.2017.0109.
4. Carmona L. The burden of musculoskeletal diseases in the general population of Spain: results from a national survey. *Annals of the Rheumatic Diseases.* 2001;60(11):1040-1045. 10.1136/ard.60.11.1040.
5. Lim CS, Mayer-Brown SJ, Clifford LM, Janicke DM. Pain is Associated with Physical Activity and Health-Related Quality of Life in Overweight and Obese Children. *Children's Health Care.* 2014;43(3):186-202. https://doi.org/10.1080/02739615.2013.837825.
6. Varni JW, Seid M, Kurtin PS. PedsQLTM 4.0: reliability and validity of the Pediatric Quality of Life InventoryTM Version 4.0 Generic Core Scales in healthy and patient populations. *Med Care.* 2001;39(8):800-812. https://doi.org/10.1097/00005650-200108000-00006.
7. Wilson IB, Cleary PD. Linking Clinical Variables with Health Related Quality of Life - A Conceptual Model of Patient Outcomes. 1995.
8. O'Sullivan PB, Beales DJ, Smith AJ, Straker LM. Low back pain in 17 year olds has substantial impact and represents an important public health disorder: a cross-sectional study. *BMC Public Health.* 2012;12(1):100. https://doi.org/10.1186/1471-2458-12-100.
9. Chandler JL, Brazendale K, Beets MW, Mealing BA. Classification of physical activity intensities using a wrist-worn accelerometer in 8-12-year-old children. *Pediatric Obesity.* 2016;11(2):120-127. https://doi.org/10.1111/ijpo.12033.
10. Farr JN, Going SB, Lohman TG, et al. Physical activity levels in patients with early knee osteoarthritis measured by accelerometry. *Arthritis & Rheumatism.* 2008;59(9):1229-1236. 10.1002/art.24007.
11. Larsson C, Ekvall Hansson E, Sundquist K, Jakobsson U. Impact of pain characteristics and fear-avoidance beliefs on physical activity levels among older adults with chronic pain: a population-based, longitudinal study. *BMC Geriatrics.* 2016;16(1). 10.1186/s12877-016-0224-3.
12. Varni JW, Burwinkle TM, Seid M, Skarr D. The PedsQL 4.0 as a health measure: feasibility, reliability, and validity. *Ambulatory Pediatrics.* 2003;3(6):329-341. https://doi.org/10.1367/1539-4409(2003)003<0329:TPAAPP>2.0.CO;2.
13. Varni JW SM, Rode CA. The PedsQL: measurement model for the pediatric quality of life inventory. *Med Care.* 1999;37(2):126-139. https://doi.org/10.1097/00005650-199902000-00003.

Supplementary material C – Sensitivity analyses

Descriptive statistics of each item for main and both sensitivity analyses

Item	Statistic	Main analysis	Pain Last week	Pain Often
**Pain intensity**				
	N	609	372	146
	Mean	3.37	3.58	4.06
	Std Deviation	1.33	1.34	1.30
	Skewness	0.50	0.29	-0.04
	Kurtosis	-0.64	-0.77	-0.78
	Median	3	3	4
**Pain interfered with normal activity**				
	N	610	376	149
	Mean	2.01	2.16	2.48
	Std Deviation	0.98	1.01	1.10
	Skewness	0.68	0.46	0.06
	Kurtosis	-0.54	-0.87	-1.31
	Median	2	2	2
**Pain sometimes stopped me from doing sport or physical activity**				
	N	608	375	148
	Mean	2.07	2.16	2.28
	Std Deviation	0.96	0.98	1
	Skewness	0.56	0.49	0.39
	Kurtosis	-0.63	-0.76	-0.89
	Median	2	2	2
**Stayed home from school because of pain**				
	N	607	373	148
	Mean	1.63	1.68	1.97
	Std Deviation	0.81	0.84	0.98
	Skewness	1.06	0.97	0.64
	Kurtosis	0.19	-0.08	-0.69
	Median	1	1	2
